# Detail data of reactive extraction of caproic acid using tri-Butyl phosphate and Sunflower and Soybean oils as diluents

**DOI:** 10.1016/j.dib.2020.105836

**Published:** 2020-06-11

**Authors:** Sourav Mukherjee, Basudeb Munshi

**Affiliations:** a Department of Chemical Engineering, National Institute of Technology, Rourkela, Odisha, India

**Keywords:** Caproic acid, Distribution co-efficient, Tbp, Reactive extraction, Sunflower, Soybean

## Abstract

Caproic acid can be produced by fermentation technology. Reactive extraction method is a promising technology for separating the acid from the carboxylic mixture in the fermenter [1], [2], [3], [4]. To achieve it, tri‑butyl phosphate (TBP) is used as the reactive extractant and sunflower and soybean oils are used as the diluents. The performance of both the physical and reactive extraction processes was analysed by different parameters like distribution coefficient, loading ratio, and extraction efficiency. To meet the purpose, concentration of caproic acid in aqueous phase was measured by doing acid-base titration by caustic solution Further, reaction equilibrium constant, stoichiometry and distribution of complex, free acid and dimer concentrations in the organic phase were analysed. The data are related to the published (https://doi.org/10.1016/j.cep.2020.107926) paper in Chemical Engineering and Processing: Process Intensification [5]. The data shown in the current article are not provided in the mentioned published paper. Moreover, data are useful for understanding the physical and chemical behavior of the caproic acid extraction process and also can be used to design the process in industrial scale.

Specifications table

**Table:** 

Subject	Filtration and Separation
Specific subject area	Mass Transfer, Reactive Extraction, Green Process, Process Intensification
Type of data	Table (.docx), figurers (image), .xlsx
How data were acquired	Concentration of caproic acid in the aqueous phase was measured by titrating against caustic solution. The acid concentration in the organic phase was estimated by mass balance method. Temperature inside the incubator shaker was measured by k-type thermocouple. The enthalpy and entropy changes of the isothermal process were estimated by data fitting method. The chemical equilibrium constant was also estimated by data fitting method. The variation of the free, dimer and complex acid concentration were calculated by solving component mass balance equations.
Data format	The collected raw data during the experiment are available in .xlsx format.
Parameters for data collection	The equilibrium concentration distribution of caproic acid in aqueous and organic phases were determined at 298, 303, 308, 313 and 318 K temperatures. The initial concentration of the acid in the aqueous phase was varied in the range of 0.00848 to 0.055 mol/L. In the aqueous phase, TBP concentration was varied from 0.366 to 1.466 mol/L.
Description of data collection	The concentration of caproic acid in the aqueous phase was measured by the acid-base titration method. Concentration of acid in organic phase was estimated by mass balance.
Data source location	Institution: National Institute of Technology, Rourkela City/Town/Region: Rourkela, Odisha Country: India
Data accessibility	With the article
Related research article	Author's name: Sourav Mukherjee, Basudeb Munshi Title: Experimental and Theoretical Analysis of Reactive Extraction of Caproic Acid by Using TBP in Green Diluents Journal: Chemical Engineering and Processing: Process Intensification Revised copy was submitted.

## Value of the data

• The experimental data can be used to examine the suitability of the extractant and green diluent for recovering caproic acid from fermentation unit. The data also help to identify the mechanism of mass transport of the caproic acid from aqueous phase to the organic phase.
• The raw data provides distribution coefficient, chemical reaction equilibrium constant, reaction stoichiometry and distribution of caproic acid in different forms in the organic phase. These data can be used to develop phase diagram of the caproic acid reactive extraction system. The graph can be used further for finding number equilibrium stages.
• These data alogn with mass balance equations can be used for designing continuous co-current and counter-current reactive extraction cascades.

## 1 Data description

The presented data in this paper corresponds to the measured concentrations of caproic acid at equilibrium state. The initial acid concentration in the aqueous phase were also presented. The concentration data were presented at five different temperature levels. The raw data are available in “RW” folder. Data using Sunflower oil and soybean oil as diluents are available in “SUN” and “SOY” folder, respectively. In “SUN” folder, physical and chemical extraction data are available in“phy-sun.xlsx” and “chem-sun.xlsx” files, respectively. Similarly, in “SOY” folder, “chem-soy.xlsx” and “phy-soy.xlsx” files, respectively contain chemical and physical extraction raw data. Data at different temperatures in the .xlsx files are available in different worksheets named by 298 K, 301 K, etc.The variations of $K_{D}^{\text{overall}}$, $K_{D}^{\text{chem}}$, complex concentration, [*HA_m_S_n_*]_*org*_, loading ratio, *z* and efficiency of the reactive extraction process (**E**%**)** with initial and equilibrium acid concentrations are tabulated in Tables 1–8. TBP concentrations and temperaturers are used as the parameter. All the tables are available in “Chemtab.docx”file.

**Table 1:** Structure of raw data (in .xlsx file) in physical extraction process.

Temperature =		

${\left[\mathrm{HA}\right]}_{\mathrm{aq}}^{0}$**mol/L_`_**	${\left[\mathrm{HA}\right]}_{\mathrm{aq}}^{\mathrm{tot}}$**(mol/L)**	${\left[\mathrm{HA}\right]}_{\mathrm{org}}^{\mathrm{tot}}$**mol/L**

**Fig. 1:**
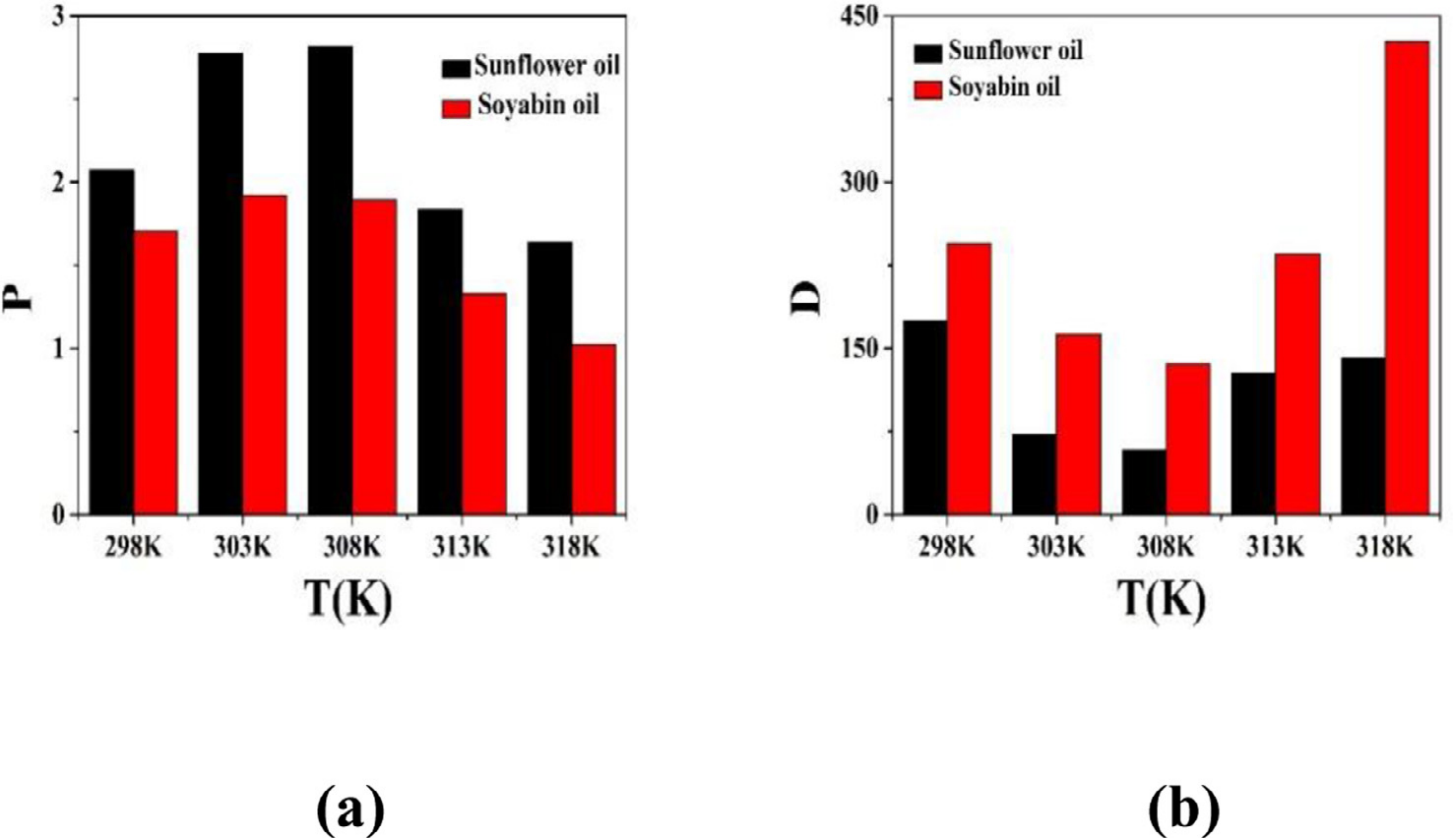
Variation of (a) Partition co-efficient and (b) Dimerization constant with temperature in the physical extraction process.

**Fig. 2:**
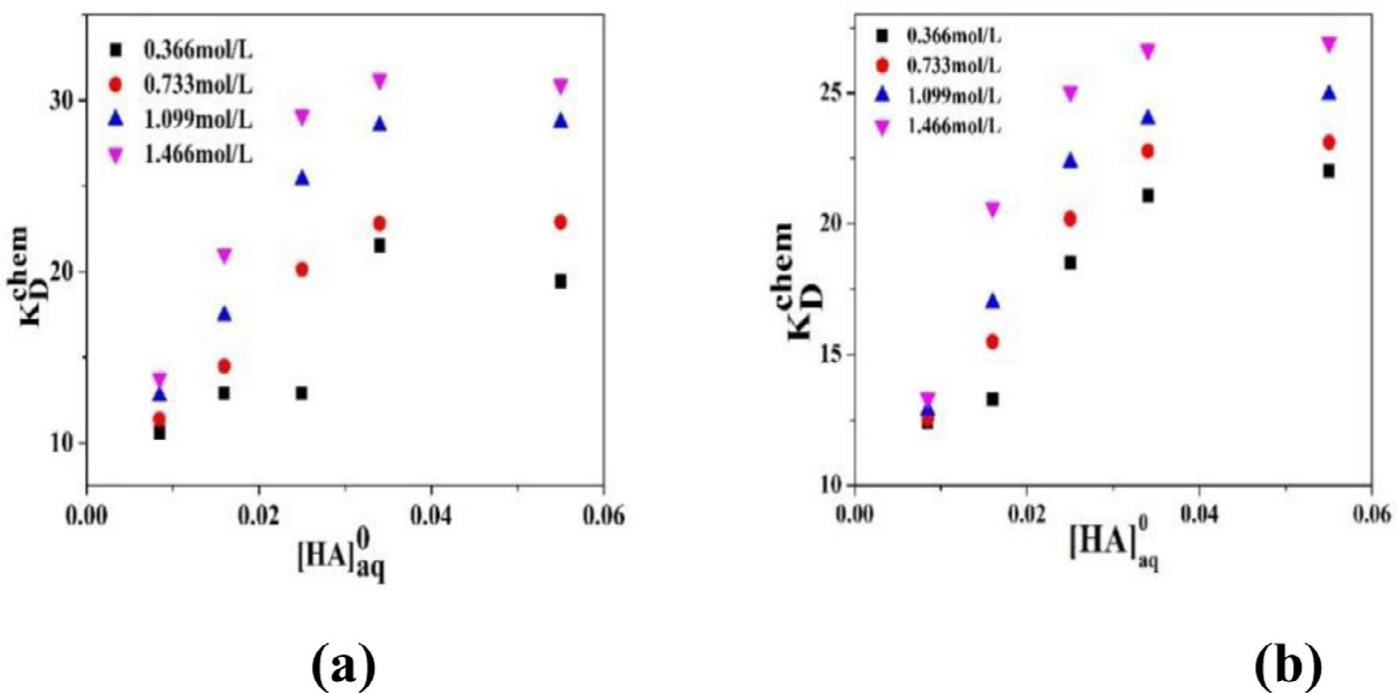
Variation of$K_{D}^{chem}$ with initial concentration of caproic acid at 298 K using (a) Sunflower oil and (b) Soybean oil as diluents. TBP concentration is varied in the range of 0.366 to 1.466 mol/L.

**Fig. 3:**
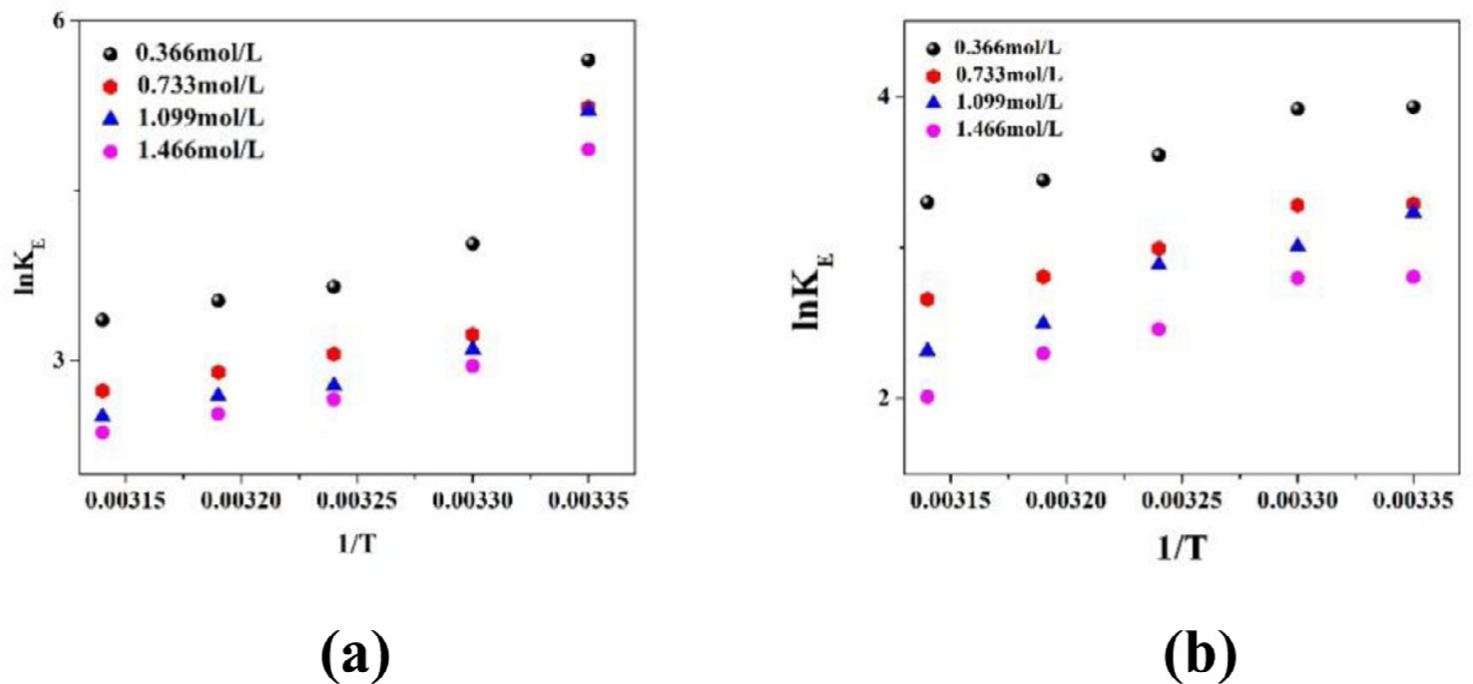
Variation of ln*K_E_*with*1/T*using (a) Sunflower oil and (b) Soybean oilas diluents. TBP concentration is varied in the range of 0.366 to 1.466 mol/L.

**Fig. 4:**
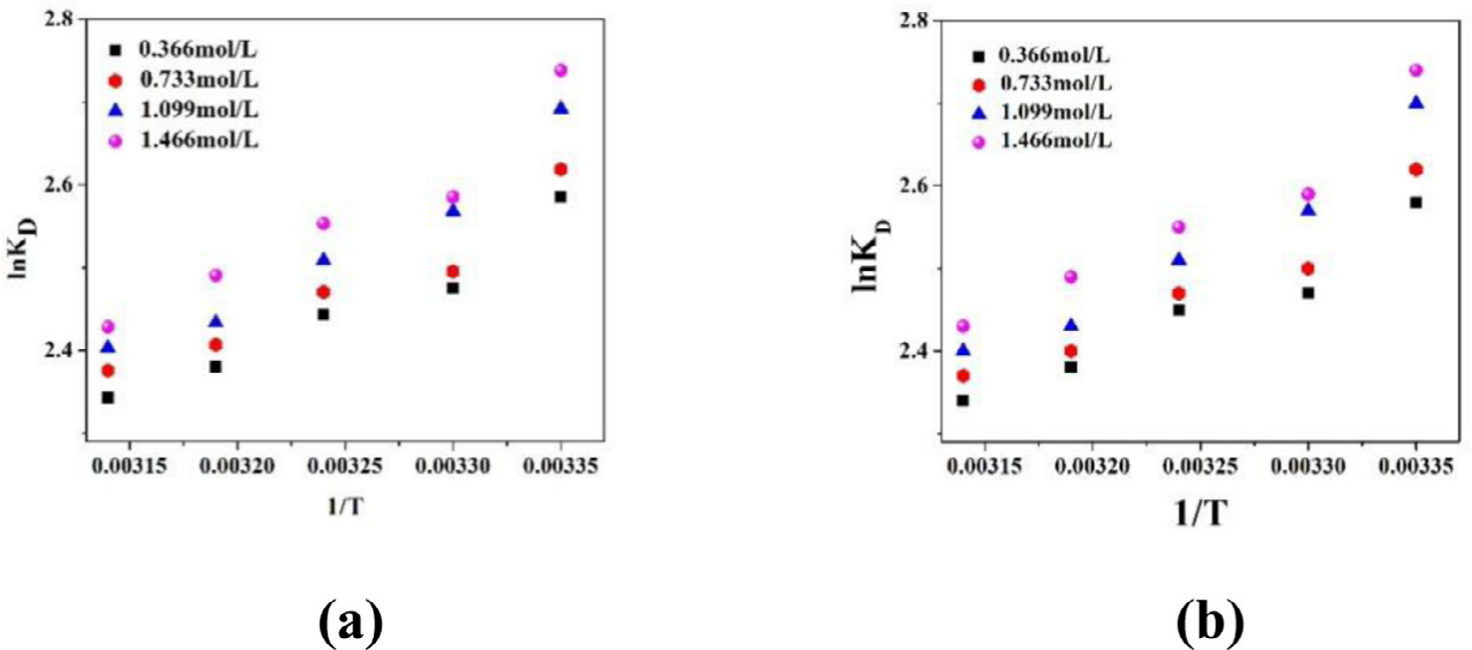
Variation of ln*K_D_* with *1/T*using (a) Sunflower oil and (b) Soybean oilas diluents. TBP concentration is varied in the range of 0.366 to 1.466 mol/L.

The variation of partition coefficient (P) and dimerization constant (D) in case of physical extraction are depicted in Fig. 1. Fig. 2 shows the graphical variation of $K_{D}^{\text{chem}}$.Thevariation of ln*K_E_* with 1/*T* is shown in Fig. 3. Fig. 4 shows the variation of ln*K_D_* with 1/*T*.

## 2 Experimental design, materials, and methods

### 2.1 Experimental design

The equilibrium reactive extraction of caproic acid from its aqueous solution was carried out by mixing the solution with organic solvent (TBP as extractant and sunflower oil or soybean oil as diluent) at a particular temperature. Complete experiment was performed for 12 h in incubator shaker to achieve the equilibrium state. Temperature was varied in the range of 298 to 318 K with 5 K increment.

### 2.2 Materials

Tri-n-Butyl Phosphate **(**Loba Chemie Pvt. Ltd, India), with assay of 99%, was used as the reactive extractant. Caproic acid (Otto Chem. Ltd., India), with assay of 99%,was used. Sunflower oil (Agro Tech Foods Ltd, India) and soybean oil(Cargill India Pvt. Ltd.) were used as diluent. NaOH (Fisher Scientific Chemicals Pvt. Ltd., India) was used for estimating caproic acid in aqueous phase using titration method. For standardizing NaOH solution, oxalic acid (Fisher Scientific, India) with assay 99%was used. Phenolphthalein (Merck Specialties Pvt. Ltd., India) was used as an indicator.

### 2.3 Methods

10 cc each of organic and aqueous phases were taken in a 100 ml conical flask and mixed for 12 h in an orbital incubator shaker. Temperature of the shaker was maintained from 298 K to 318 K. After 12 h, the mixture was allowed to settle in a separator for two hours maintaining the same experimental temperature. Further, the aqueous was titrated against caustic solution to know the equilibrium concentration of caproic acid in the aqueous phase. The concentration of caustic solution was measured by primary standard oxalic acid. The total concentration of caproic acid in the organic phase was estimated by mass balance. The distributions of $K_{D}^{\text{overall}}$, $K_{D}^{\text{chem}}$, [*HA_m_S_n_*]_*org*_, *z* and E% were estimated from the measured caproic acid concentration distributions using the different relations given in the submitted paper with manuscript number CEP_2019_1502 in Chemical Engineering and Processing: Process Intensification.

Tables 2 and 3.

**Table 2:** Structure of raw data (in .xlsx file) in chemical extraction process.

Temperature =			

[**S**]_0_**mol/L**	${\left[\mathrm{HA}\right]}_{\mathrm{aq}}^{0}$**mol/L_`_**	${\left[\mathrm{HA}\right]}_{\mathrm{aq}}^{\mathrm{tot}}$**(mol/L)**	${\left[\mathrm{HA}\right]}_{\mathrm{org}}^{\mathrm{tot}}$**mol/L**

**Table 3:** Structure of data in Tables 1 to 8 of “Table of Chemical Extraction data.docx” file.

**[S]**_**0**_**mol/L**	${\left[\mathrm{HA}\right]}_{\mathrm{aq}}^{0}$ mol/L_`_	${\left[\mathrm{HA}\right]}_{\mathrm{org}}^{\mathrm{tot}}$ mol/L	$K_{D}^{\mathrm{overall}}$	$K_{D}^{\mathrm{chem}}$	[***HA***_***m***_***S***_***n***_]_***org***_	***z***	**E%**

## Declaration of Competing Interest

The authors declare that they have no known competing financial interests or personal relationships which have, or could be perceived to have, influenced the work reported in this article.
